# Female Scent Signals Enhance the Resistance of Male Mice to Influenza

**DOI:** 10.1371/journal.pone.0009473

**Published:** 2010-03-01

**Authors:** Ekaterina A. Litvinova, Elena P. Goncharova, Alla M. Zaydman, Marina A. Zenkova, Mikhail P. Moshkin

**Affiliations:** 1 Laboratory of Physiological Adaptation of Animals, Institute of Systematics and Ecology of Animals, Siberian Branch, Russian Academy of Sciences, Novosibirsk, Russia; 2 Laboratory of Nucleic Acids Biochemistry, Institute of Chemical Biology and Fundamental Medicine, Siberian Branch, Russian Academy of Sciences, Novosibirsk, Russia; 3 Department of Histology, Novosibirsk Institute of Traumatology, Novosibirsk, Russia; 4 Department of Animal Genetic Resources, Institute of Cytology and Genetics, Siberian Branch, Russian Academy of Sciences, Novosibirsk, Russia; Institut Pasteur Korea, Republic of Korea

## Abstract

**Background:**

The scent from receptive female mice functions as a signal, which stimulates male mice to search for potential mating partners. This searching behavior is coupled with infection risk due to sniffing both scent marks as well as nasal and anogenital areas of females, which harbor bacteria and viruses. Consideration of host evolution under unavoidable parasitic pressures, including helminthes, bacteria, viruses, etc., predicts adaptations that help protect hosts against the parasites associated with mating.

**Methods and Findings:**

We propose that the perception of female signals by BALB/c male mice leads to adaptive redistribution of the immune defense directed to protection against respiratory infection risks. Our results demonstrate migration of macrophages and neutrophils to the upper airways upon exposure to female odor stimuli, which results in an increased resistance of the males to experimental influenza virus infection. This moderate leukocyte intervention had no negative effect on the aerobic performance in male mice.

**Conclusions:**

Our data provide the first demonstration of the adaptive immunological response to female odor stimuli through induction of nonspecific immune responses in the upper respiratory tract.

## Introduction

The scent of female mice as a signal for reproduction stimulates male mice to search for a breeding partner [1]–[3]. In turn, searching behavior increases the risk of respiratory infections in male mice due to sniffing the contaminated female fecal and urinary marks, which harbor many infection agents [4]–[6]. Naso-nasal sniffing when the partner is located adds an infection risk from air-borne bacteria and viruses.

The most significant systems involved in the defense against respiratory infections are epithelia, mucosa, and mucosa-associated leukocytes, which not only provide a passive barrier function, but also actively contribute to the innate immune system response [7]–[8]. In particular, the recruitment of neutrophils and mononuclear phagocytes to the alveolar air space limits viral and bacterial interventions [9]. Recently, we have found that counts of macrophages, neutrophils, and eosinophils in bronchoalveolar lavages (BAL) are significantly higher in male mice given a daily portion of soiled bedding from female cages. This is a natural sexual signal compared with the males kept in isolation from female odors [10]. Since immunocompetent cells are the major effectors of the protective inflammatory response to respiratory infections [8], [11], we can expect both positive and negative consequences of the exposure to female chemical signals. The scent-induced leukocyte recruitment provides an anticipatory adaptation to infection risks. On the other hand, migration of the inflammatory cells into the alveolar air space can influence pulmonary capacity, which declines during an inflammation due to lung edema [12]–[14]. This can lead to reduction of the capability to mobilize energy in response to social and climatic challenges. One of the best approaches for assessment of the cardiopulmonary efficiency (aerobic performance) is based on measurement of the maximum oxygen consumption (MOC) as an indicator of physical fitness in humans and mice [15]–[16].

For testing both predictions, we studied male mice kept either with or without female odors. In these groups of mice, we assessed leukocyte recruitment into upper airways by histological examination of lung tissue, estimated the resistance of to influenza virus as an example of respiratory infection, and measured MOC in as an indicator of aerobic performance. We used mouse-adapted influenza virus A/WSN/33 (H1N1). Some human-derived strains of influenza viruses replicated efficiently in the mouse lungs without prior host adaptation [17]–[18]. Influenza does not occur in feral populations *Mus musculus* due to high virulence and low contagiousness [19]–[20].

## Methods

### Animals and Housing Conditions

We obtained 8–10-week-old inbred BALB/c mice from the stock of the Institute of Clinical Immunology (Siberian Branch, Russian Academy of Medical Sciences, Novosibirsk, Russia). All male (*n* = 106) and female (*n* = 25) mice were housed (4–6 weeks) in single-sex groups of five or six animals except for 15 min required for testing the maximum aerobic performance. We maintained male and female groups in standard plastic cages (35×21×9 cm) with sawdust (wooden flakes) as nesting material. We provided food pellets (BioPro, Novosibirsk) and water *ad libitum*. Mice were maintained at 22–24°C under 12∶12 light-dark cycle with lights off at 1400 hrs.

In each cage, males were individually marked by strokes on the tail made with surgical skin markers. Every 5 days, animals were placed into a clean cage with fresh sawdust. Mice were used in experiments within 2 weeks after arriving. We randomly divided male mice into two groups: males exposed to female scent (*n* = 52) and the males isolated from female chemical signals (*n* = 54). The female mice and two groups of males were kept in separate animal rooms with independent ventilation.

### Female Bedding Treatment

The bedding (wooden flakes) from the cages with mature BALB/c females was used for exposing males to female odors, as it is the most natural sexual signals for mice. Using five cages with five females per cage, we collected female bedding soiled during the previous 5 days, exposing males to a new portion (5 ml) of soiled female bedding each day. Males in the treatment with no exposure to females were given a similar sample of clean bedding. We added new portions of bedding daily approximately 1 h before lights off.

According to the recommendation of Vandenberg [21], we provided female mice with 2–3 g of soiled bedding from male cages to regularize their estrus cycles. Examination of vaginal smears 2 weeks after the scent treatment demonstrated that at least 30% of the female mice were in estrus. Thus, each 5-day-old bedding sample from the female cages was contaminated with urine and feces of receptive female mice.

### Infection with Influenza Virus

We used male BALB/c mice in the experiment as one of the inbred mouse strains most susceptible to influenza virus A/WSN/33 (H1N1). The virus was obtained from the collection of the Ivanovsky Institute of Virology (Russian Academy of Medical Sciences, Moscow, Russia). Human influenza virus A/WSN/33 (H1N1), carrying a specific change in the M1 protein, causes a lethal pneumonia in BALB/c mice [19]. Virus was grown in embryonic chicken eggs. We harvested allantoic fluid, containing the virus and stored it in aliquots at –80°C. Prior to application, the virus stock (5×10^7^ FFU (focus-forming units)/ml) was serially diluted tenfold with cold phosphate-buffered saline (PBS). Males were infected intranasally (under ether anesthesia) with the influenza virus A/WSN/33 (H1N1) in 50 µl of PBS. We used five serially diluted doses that contained from 2.5×10^1^ to 2.5×10^5^ FFU. Mice were weighed every two days for 10 days after infection with influenza virus. Mortality cases were recorded daily 1 h before lights off during 3 weeks until full recovery of the surviving male mice. For one week prior to the experimental infection and during 3 weeks after infection, BALB/c males were exposed to either female-soiled bedding (*n* = 41) or fresh wooden flakes (*n* = 41).

### Leukocyte Aggregates in the Lung Tissue

We made histological preparations of the lung tissue harvested from the additional groups of uninfected male mice that were either exposed (*n* = 3) or not exposed (*n* = 5) to female bedding during 1 week. Lung samples were fixed in 10% buffered formalin. Histological sections (4 µm) of the lung samples were stained with hematoxylin and eosin (HE). The number of leukocyte aggregates, their size, and the signal intensity in grey scale were determined in each section. We calculated the number of leukocytes per section using a regression equation [22].

### Aerobic Performance

Maximum oxygen consumption was assayed in uninfected male mice, which were exposed to either female bedding (*n* = 8) or fresh wooden flakes (*n* = 8) during 1 week before the measurement. The mice were exposed to HELOX (20% O_2_: 80% He) to stimulate energy metabolism [23]. During this test, male mice were kept singly in respirometry boxes (volume, 0.5 L). The animals were monitored in a closed respirometer maintained at 10°C and aerated by a circuit pump with HELOX for 15 min. Dry NaOH was used for CO_2_ binding. During each minute, except for 5th and 10th minutes required for O_2_ supply, the HELOX circulation was shut off for 10 s to measure current oxygen consumption (VO_2_) by a drop of pressure in an electronic manometer. The VO_2_ was adjusted to standard conditions (0°C, 1.01×10^6^ Pa). The individual peak of oxygen consumption was regarded as MOC.

### Statistical Analysis

Differences in the mortality rates between scent treated and untreated infected males was calculated using a chi-square test. We used two-way ANOVA to determine the influence of female scent and the dose of virus on the maximal weight loss in infected male mice. LD_50_ was calculated according to Reed and Muench [24]. The dose-dependent maximal weight loss was processed by the least significant difference (LSD) test. We used Student's *t*-tests to compare LD_50_, maximal weight loss, and MOC for the two treatment conditions. Differences in the number of leukocyte aggregates and leukocyte counts in lung tissue were assessed by a Mann–Whitney *U* test. We used repeated measures ANOVA for comparing the VO_2_ dynamics in scent treated and untreated males during their exposure to the HELOX environment. All data are expressed as the mean ± SE.

### Ethics Statement

All animals were handled in strict accordance with good animal practice as defined by the relevant national and/or local animal welfare bodies, and all manipulations with animals were approved by the appropriate committee according to “The Guidelines for Manipulations with Experimental Animals” (order of the Presidium of the Russian Academy of Sciences of April 02, 1980 no. 12000-496).

## Results

### Resistance to Influenza

**Figure 1:**
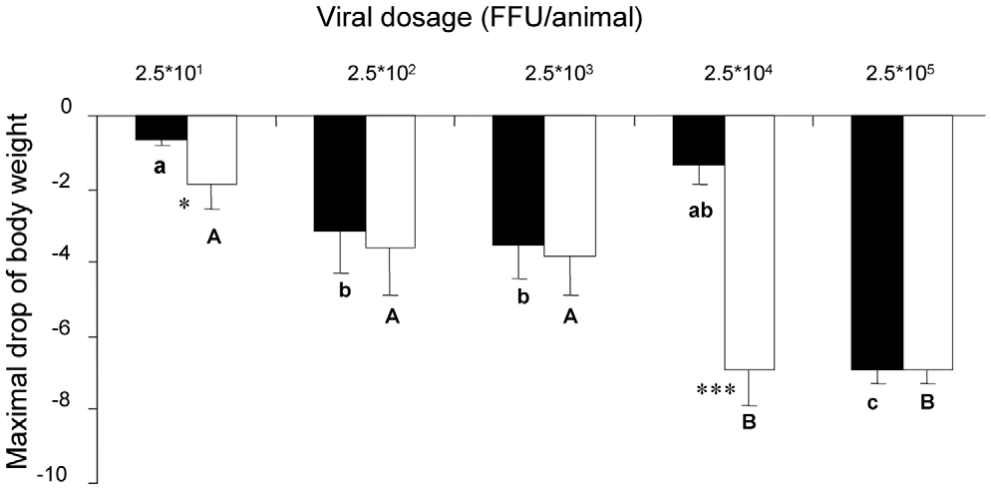
Maximal decrease in the weight of male mice during 3 weeks after infection with influenza. BALB/c male mice unexposed (white columns) and exposed (black columns) to the female bedding (mean ± SE). Capital letters mark statistically significant differences of means in the males not exposed to female bedding and small letters mark statistically significant differences of the means in males exposed to female bedding (LSD test, *p*<0.05). * *p*<0.05, *** *p*<0.001, compared with the scent exposed mice (Student's *t*-test).

**Figure 2:**
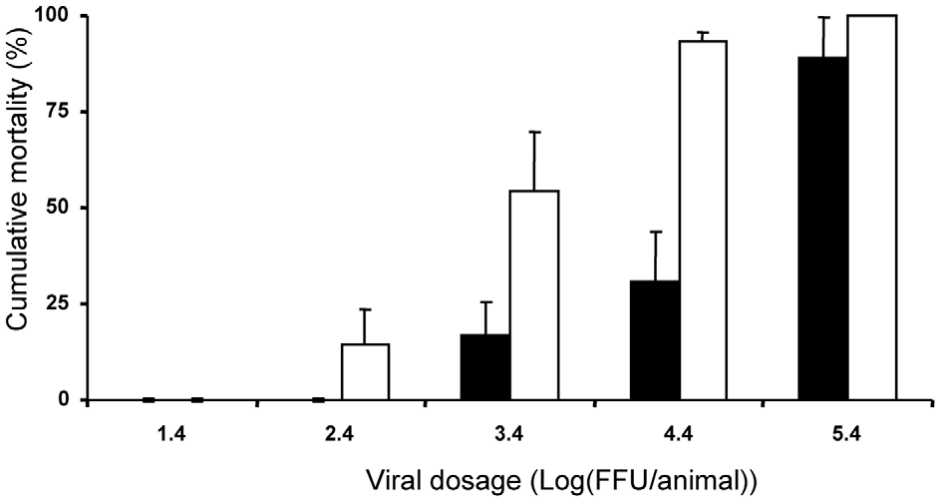
Cumulative mortality after infection with influenza. BALB/c male mice not exposed (white dots) and exposed (black dots) to female bedding (mean ± SE). LD_50_ in the males exposed to female bedding (4.73±0.30 log FFU) was significantly higher as compared with the males isolated from female bedding (3.28±0.35 log FFU, *t* = 2.84, *p*<0.01).

The decrease in body mass depended on the dose of intranasally applied influenza virus (*F*_4,76_ = 6.97, *p*<0.001). The weight loss was more pronounced in the males kept in isolation from females (*F*_1,76_ = 7.80, *p*<0.01), especially under a submaximal dose of the virus (Fig. 1). The total mortality rate was lower in males exposed to female bedding (8 of 41) as compared with the male mice not exposed to the female bedding (19 of 41 mice, χ^2^ = 5.52, *P* = 0.019). The males exposed to female bedding displayed a higher LD_50_ than the males isolated from females (Fig. 2).

### Lung Histology

**Figure 3:**
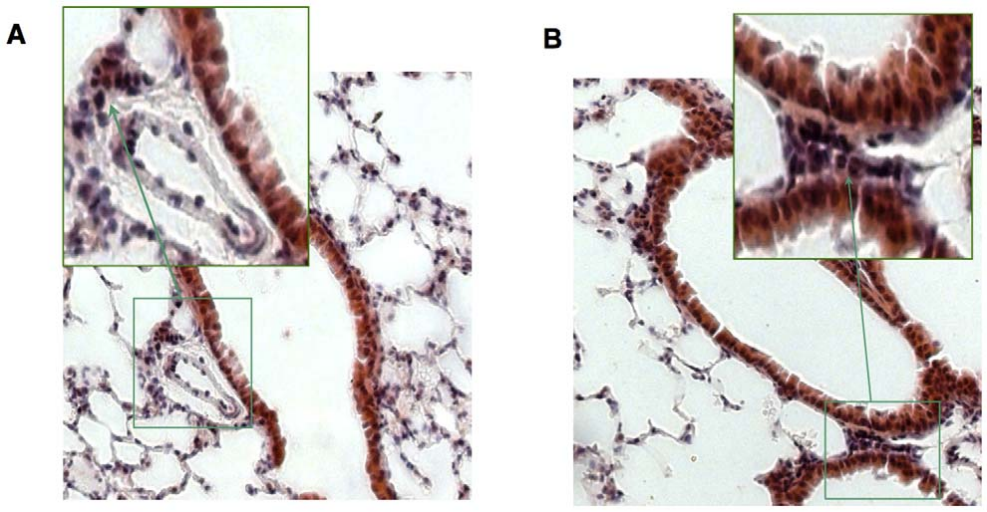
Leukocyte aggregates in the lung tissue. (**A**) Male BALB/c mice not exposed to female bedding. (**B**) Male BALB/c mice exposed to female bedding. Light microscopy (magnifications ×100 and ×200).

Examination of histological preparations of the lung tissue harvested from uninfected male mice kept in different scent environments displayed significant differences: the intervention of leukocytes into the peribronchial and perivascular areas was more pronounced in male mice exposed to female bedding as compared with the males kept in isolation from females (Fig. 3). The total number of leukocytes per histological section was significantly higher in males exposed to female soiled bedding (12268.68±1770.10, *n* = 5) than in the males isolated from females (3787.51±2677.14, *n* = 3; *p*>0.05, Mann–Whitney test). However, the number of leukocyte aggregates and average number of leukocytes per aggregate did not differ between the two groups (Table 1).

**Table 1:** The number of leukocyte aggregates per histological section: the average number of leukocytes per aggregate and total number of leukocytes per section in male BALB/c mice not exposed and exposed to female bedding (mean ± SE).

Group of male mice	Number of leukocyte aggregates	Number of leukocytes per aggregate	Number of leukocytes per section
Not exposed to female bedding (*n* = 3)	31.17±19.95	109.54±10.10	3787.51±2677.14
Exposed to female bedding (*n* = 5)	93.70±16.45	133.17±4.52	12268.68±1770.10
Mann–Whitney test	Z = 1.64*p* = 0.10	Z = 1.64*p* = 0.10	Z = 2.24*p* = 0.03

### Aerobic Performance

**Figure 4:**
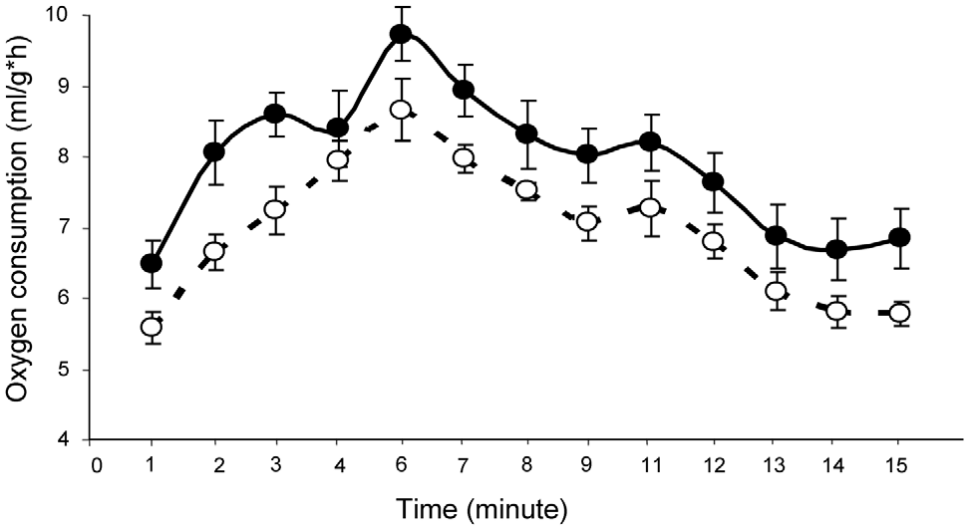
Aerobic performance. Oxygen consumption during 15-min exposure to HELOX (mean ± SE) by the male mice not exposed (white dots) and exposed (black dots) to female bedding (repeated measures ANOVA *F*_1,14_ = 5.46, *p* = 0.035). MOC was similar in the males exposed (*n* = 8, 9.73±0.39) and not exposed (*n* = 8, 9.00±0.29) to female bedding.

The cold-induced MOC did not differ between the males exposed (9.73±0.39, *n* = 8) and not exposed (9.00±0.29, *n* = 8) to female bedding (*p*>0.05, Student's *t*-test). However, oxygen consumption during a 15 min exposure to HELOX was higher at all time points in the scent treated males as compared with the males isolated from female chemical signals (Fig. 4). Repeated measures ANOVA confirmed a statistically significant effect of female odors on oxygen consumption (*F*_1,14_ = 5.46, *p* = 0.035), as well as on the time points of oxygen consumption dynamics (*F*_12,168_ = 41.34, *p*<0.001).

## Discussion

In addition to the well known induction of the neuroendocrine regulation of spermatogenesis and mating behavior by female signals [2], [3], [25], our work demonstrates that such signals play a role in increasing the resistance to influenza virus, which was used as an available respiratory pathogen. This dual effect of female odors can compensate for the risk of respiratory infections, which accompanies the pheromone-induced searching behavior and olfactory assessment of a mating partner. The phenomenon is causally explainable by an increase in the number of immunocompetent cells in lung tissue of male mice exposed to female soiled bedding. In our previous study, we found significantly more macrophages, neutrophils, and eosinophils in the lung lavage fluids from males exposed to female soiled bedding as compared with males maintained without exposure to male chemical signals [10]. Both neutrophils and macrophages are major effectors of the nonspecific immune defense against respiratory infections [7], [8], [26], and their migration to the upper airways increases resistance to experimental pneumonia [27]. This may help explain the enhanced resistance to influenza in male mice observed after exposure to the female- soiled bedding.

We did not find any special studies into the leukocyte recruitment induced by sexual pheromones or the pheromone-dependent neuroendocrine mechanism except for experimental evidence of female scent- or testosterone-induced decrease in circulating monocytes [10], [28] and the hypothesis on androgen-induced reallocation of the immune defense to skin and other peripheral areas [29]. We also cannot reject non-pheromonal explanations of the leukocyte recruitment to the upper airways, such as the effect of bacterial components inhaled from contaminated soiled bedding during sniffing. Regardless, bedding contaminated with bacteria is a significant component of the natural sexual signals in mice. In addition, some bacterial compounds are recognized by formyl peptide receptors (FPRs) in vomeronasal olfactory epithelium [30]. In our study, we kept males in single-sex groups, and they could sniff bedding soiled by other males; nevertheless, pheromonal and bacterial components of this bedding did not increase their resistance to influenza virus to the level that we observed in the males exposed to female odors.

The leukocyte migration to lung tissue is a common manifestation of respiratory tract inflammation, which entails a decline in the efficiency of both lung ventilation and aerobic performance [31], [32]. However, the sizes of the leukocyte aggregates in our study varied from 11 to 1029 cells per aggregate, which is considerably smaller than reported for a typical inflammatory infiltration [33], [34]. A direct assessment of the functional reserves of the cardiorespiratory system showed that, in contrast to the predicted negative respiratory effect of leukocyte migration into the lungs, the scent treated males demonstrated a higher VO_2_ under cooling in HELOX than males isolated from female chemical signals. However, the maximal aerobic performance was similar in both male groups.

Thus, our data are the first to demonstrate the migration of leukocytes in response to natural mating-related olfactory signals. This provides for an anticipatory adaptation of male mice to potential risks of respiratory infections and, concurrently, does not reduce their aerobic performance.
